# The Effects of Valvular Heart Disease on Atrial Conduction During Sinus Rhythm

**DOI:** 10.1007/s12265-019-09936-8

**Published:** 2019-11-26

**Authors:** Lisette J. M. E. van der Does, Eva A. H. Lanters, Christophe P. Teuwen, Elisabeth M. J. P. Mouws, Ameeta Yaksh, Paul Knops, Charles Kik, Ad J. J. C. Bogers, Natasja M. S. de Groot

**Affiliations:** 1 Department of Cardiology, Erasmus Medical Center Rotterdam, Dr. Molewaterplein 40, 3015 GD Rotterdam, The Netherlands; 2 Department of Cardiothoracic Surgery, Erasmus Medical Center Rotterdam, Rotterdam, The Netherlands

**Keywords:** Valvular heart disease, Mapping, Sinus rhythm, Conduction, Atrial fibrillation

## Abstract

Different arrhythmogenic substrates for atrial fibrillation (AF) may underlie aortic valve (AV) and mitral valve (MV) disease. We located conduction disorders during sinus rhythm by high-resolution epicardial mapping in patients undergoing AV (*n* = 85) or MV (*n* = 54) surgery. Extent and distribution of conduction delay (CD) and block (CD) across the entire right and left atrial surface was determined from circa 1880 unipolar electrogram recordings per patient. CD and CB were most pronounced at the superior intercaval area (2.5% of surface, maximal degree 6.6%/cm^2^). MV patients had a higher maximal degree of CD at the lateral left atrium than AV patients (4.2 vs 2.3%/cm^2^, *p* = 0.001). A history of AF was most strongly correlated to CD/CB at Bachmann’s bundle and age. Although MV patients have more conduction disorders at the lateral left atrium, disturbed conduction at Bachmann’s bundle during sinus rhythm indicates the presence of atrial remodeling which is related to AF episodes.

## Introduction

Valvular heart disease predisposes to the occurrence of atrial tachyarrhythmias. Significant stenosis or regurgitation of heart valves has important hemodynamic effects on the atria that lead to structural atrial remodeling. In situations of pressure or volume overload, the increased pressure on the atrial wall causes atrial dilation [1]. Chronic mechanical stretch initiates pathways that produce fibrosis and alter myocyte coupling and function [2, 3]. Consequently, electrical conduction in the atria becomes disrupted and can result in areas with slow and discontinuous conduction which may act as a substrate for tachyarrhythmias. The incidence of atrial fibrillation (AF) is higher in patients with mitral valve (MV) disease (26–54%) than in patients with aortic valve (AV) disease (10–13%), most likely due to higher left atrial loads in MV disease [4, 5]. Therefore, different types of atrial remodeling may take place in AV and MV disease.

It has been previously demonstrated that structural remodeling and not electrical remodeling is the major contributor to development of AF in models of chronic atrial stretch [3]. The electrophysiological disturbances due to structural alterations in patients with valvular heart disease may also be identifiable during sinus rhythm. The purpose of this study is first to identify differences in the amount and distribution of high-resolution conduction disorders during sinus rhythm between patients with AV and MV disease. Secondly, we aim to determine if predilection sites of conduction disorders during sinus rhythm exist in patients with valvular heart disease who have developed clinical AF. A high-resolution epicardial mapping approach was performed to locate atrial areas with conduction abnormalities and identify the differences in conduction disorders between patients with AV and MV disease requiring surgical treatment.

## Methods

### Study Population

Patients of 18 years and older undergoing AV (mainly for aortic stenosis) or MV (for mitral regurgitation) surgery with or without coronary artery bypass grafting participated in this study. The study is part of the QUASAR study [6] that was approved by the local ethics committee and all patients gave informed consent prior to surgery.

### Study Procedure

**Fig. 1:**
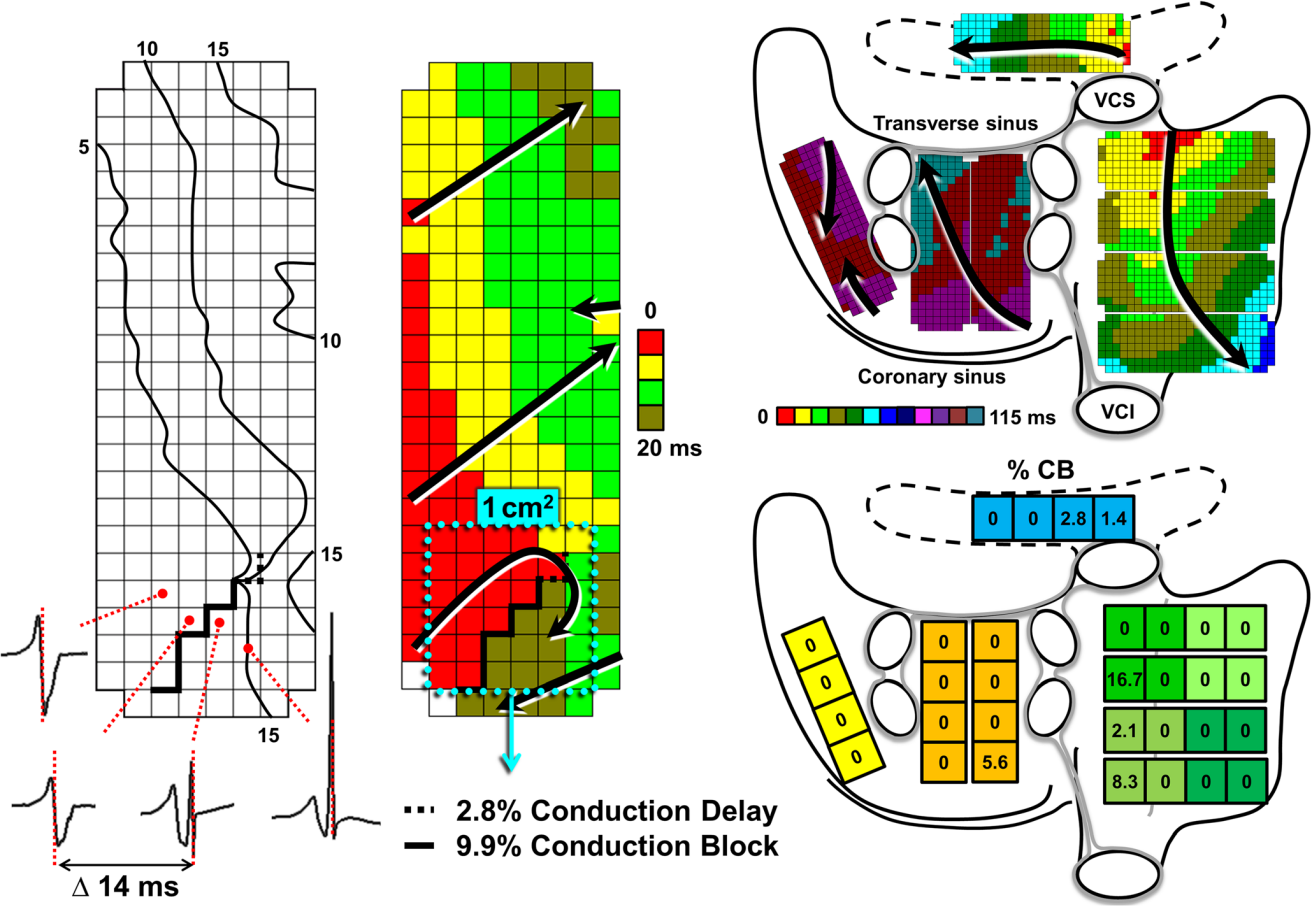
Intraoperative epicardial mapping scheme and data analysis. Left: Example of an isochronal and activation map demonstrating the analysis of conduction delay (CD) and conduction block (CB) on a high-resolution scale. An isochronal map with electrograms at the site of CB and the activation map demonstrate differences ≥ 12 ms in activation times between adjacent electrodes (interelectrode distance 2 mm). Thick (dashed) black lines indicate sites with CD (dashed line, ∆7–11 ms) and CB (solid line, ∆ ≥ 12 ms). In an area of 1 cm^2^, the number of lines with CD and CB are 2.8% and 9.9% of the total number of lines within the 1-cm^2^ quadrant. Right: High-resolution activation maps are recorded at all epicardial accessible sites: the right atrium, Bachmann’s bundle, posterior left atrial wall between the pulmonary veins and lateral left atrium including the appendage. The entire atrial surface in this patient is activated in 115 ms. The total recording area divided into 1 cm2 quadrants represents the amount of CD and CB at specific sites; the amount of CB is depicted in this example. The quadrants are assigned to 7 atrial areas: superior and inferior intercaval right atrium, the superior and inferior lateral right atrium (shades of green), Bachmann’s bundle (blue), posterior left atrium (orange) and lateral left atrium (yellow). For Bachmann’s bundle, the average degree of CB in this example is 1.0%, and the maximal degree of CB is 2.8%. VCS, vena cava superior; VCI, vena cava inferior

During surgery and prior to cardiopulmonary bypass, epicardial mapping was performed of the entire atrial surface with an electrode array containing 128 or 192 unipolar electrodes with an electrode diameter of 0.65 or 0.45 mm and inter-electrode spacing of 2 mm (GS Swiss PCB AG, Küssnacht, Switzerland). Five-second recordings of sinus rhythm were sequentially acquired from all epicardial accessible sites at the right atrium, left atrium, and Bachmann’s bundle. Patients that were in AF at the start of the procedure were electrically converted to sinus rhythm. A temporary pacemaker wire stitched to the terminal crest served as a reference signal during the recordings. The mapping procedure and mapping sites were previously described in detail [7]. Mapping locations are also illustrated in the upper right panel of Fig. 1. In case of the 128-electrode mapping array, each 192-array location consisted of two 128-array recordings. Unipolar electrograms were stored on hard disk after amplification, filtering (bandwidth 0.5–400 Hz), sampling (1KHz), and analogue to digital conversion (16 bits).

### Mapping Data Analysis

Atrial deflections were detected automatically by marking of the steepest negative deflection with a minimal slope of 80 mV/s and signal to noise ratio > 4 for each recorded electrogram in order to construct activation maps [6]. The marked electrograms and activation maps were thereafter checked and manually corrected if necessary. Aberrant and premature atrial beats were excluded from analysis. Sinus rhythm activation maps were used to determine the incidence of conduction delay (CD) and conduction block (CB) on a high-resolution scale (left panel, Fig. 1). CD and CB were defined as a difference in activation time between adjacent electrodes of, respectively, 7–11 ms and ≥ 12 ms (translating to an effective conduction velocity of 19–28 cm/s and ≤ 18 cm/s) [8]. The amount of CD and CB was calculated as a percentage per 1 cm^2^ quadrants. Figure 1 illustrates the subdivision of the entire mapping surface of each patient in 1 cm^2^ quadrants in which the percentages of CD and CB were determined.

### Atrial Distribution

The prevalence and degree of CD and CB in the patient groups were assessed separately for 7 atrial areas: 4 areas at the right atrium (the superior (1) and inferior (2) intercaval/ terminal crest area and the superior (3) and inferior (4) lateral right atrium), Bachmann’s bundle (5), posterior left atrium between the pulmonary veins (6), and the lateral left atrium/left atrial appendage (LAA) (7) (Fig. 1, right lower panel). The prevalence of CD/CB per area is defined as the percentage of patients with presence of CD or CB in that area. For each area in every patient, the average degree of CD and CB was determined (average percentage CD/CB of the 1 cm^2^ quadrants in that area) and also the maximal degree of CD and CB in 1 cm^2^ (CD/CB of the 1cm^2^ quadrant with the highest amount of CD/CB).

### Statistical Analysis

Categorical clinical characteristics between the AV and MV group were compared with Chi-square or Fisher’s exact tests. Continuous clinical data and electrophysiological data were not normally distributed and differences between the AV and MV group were evaluated with Mann-Whitney *U* tests. Electrophysiological mapping data are presented as median (minimum-maximum) values. To evaluate differences between areas, the area with the highest average and maximal degree of CD/CB was identified for each patient and the total incidences of highest amount of CD/CB were determined for each area. Differences in the incidence distributions between areas were first evaluated with the overall Cochran’s *Q* test. Subsequently, McNemar tests were applied for a pair-wise comparison of each of the areas. We applied Bonferroni correction to adjust for inflation of type I error with repeated tests. A *p* value ≤ 0.05/7 was considered statistically significant for the comparison of patient groups (AV vs MV) within the 7 areas, whereas for the inter-areal comparisons, a *p* value threshold of ≤ 0.05/21 was used. The rank-biserial correlation coefficient (skewed continuous/ordinal data) and Phi coefficient (binary data) were determined to quantify the correlation between electrophysiological/clinical variables and a history of AF. Binary logistic regression analysis was performed to relate electrophysiological and clinical variables with incidence of postoperative AF. All analyses were performed with IBM SPSS Statistics version 21 (IBM corp., Armonk, NY).

## Results

### Clinical Characteristics

Epicardial high-resolution mapping during sinus rhythm was performed in 139 patients who underwent cardiac surgery for valvular heart disease. There were 89 (64%) male and 50 (36%) female patients with a median age of 70 (IQR = 62–75) years. MV surgery was performed in 54 (39%) patients and 85 (61%) patients had valvular cardiac surgery for only AV disease. A total of 66 (47%) patients had significant coronary artery disease and 38 (27%) had a history of AF. All clinical characteristics are demonstrated in Table 1. Patients with MV disease presented more often with left atrial enlargement, left ventricular dysfunction, and AF prior to surgery; hypertension was more often observed in AV disease.

**Table 1:** Clinical characteristics

	Total	AV patients	MV patients	*P* value
	N(%)	N(%)	N(%)	
No. of patients	139	85	54	
Age, years [IQR]	70 [12]	69 [13]	70 [14]	0.641
Male gender	89 (64)	59 (69)	30 (56)	0.097
Hypertension	67 (48)	48 (57)	19 (35)	0.014
Hypercholesterolemia	29 (21)	18 (21)	11 (20)	0.909
Diabetes mellitus	22 (16)	15 (18)	7 (13)	0.461
Coronary artery disease	66 (48)	43 (51)	23 (43)	0.358
Peripheral vascular disease	7 (5)	4 (5)	3 (6)	1.000
Atrial fibrillation	38 (27)	17 (20)	21 (39)	0.015
Valvular disease*				
Aortic stenosis	81 (58)	74 (87)	7 (13)	
Aortic regurgitation	26 (19)	18 (21)	8 (15)	
Mitral stenosis	5 (4)	0	5 (9)	
Mitral regurgitation	54 (39)	0	54 (100)	
Tricuspid regurgitation	13 (9)	0	13 (24)	
Left ventricular function				0.019
Normal	103 (74)	70 (82)	33 (61)	
Mild dysfunction	25 (18)	11 (13)	14 (26)	
Moderate dysfunction	11 (8)	4 (5)	7 (13)	
Severe dysfunction	0	0	0	
Left atrial enlargement (> 45 mm)	40 (29)	11 (13)	29 (54)	<0.001

*Valvular heart disease for which corrective surgery was performed. *AV*, aortic valve; *MV*, mitral valve; *IQR*, interquartile range

### Mapping Data

The median number of electrogram recordings of the entire atrial surface acquired by high-resolution mapping was 1880 (945–2356) per patient. Quadrants containing < 50% electrogram recordings were excluded from analysis, which resulted in exclusion of 2.6% of the 4644 quadrants in total, and 32 (18–42) quadrants per patient were analyzed for presence of CD and CB. Median cycle length during mapping of sinus rhythm was not different between AV and MV patients (786 ms (473–1735) and 784 ms (533–1178), *p* = 0.28). Cardioversion was performed before mapping of sinus rhythm in 16 patients (42%) with a history of AF. However, there were no differences in the amount of conduction disorders between patients with AF who received a cardioversion and those who were spontaneously in sinus rhythm.

### Overall Conduction Delay and Block in Valvular Heart Disease

**Fig. 2:**
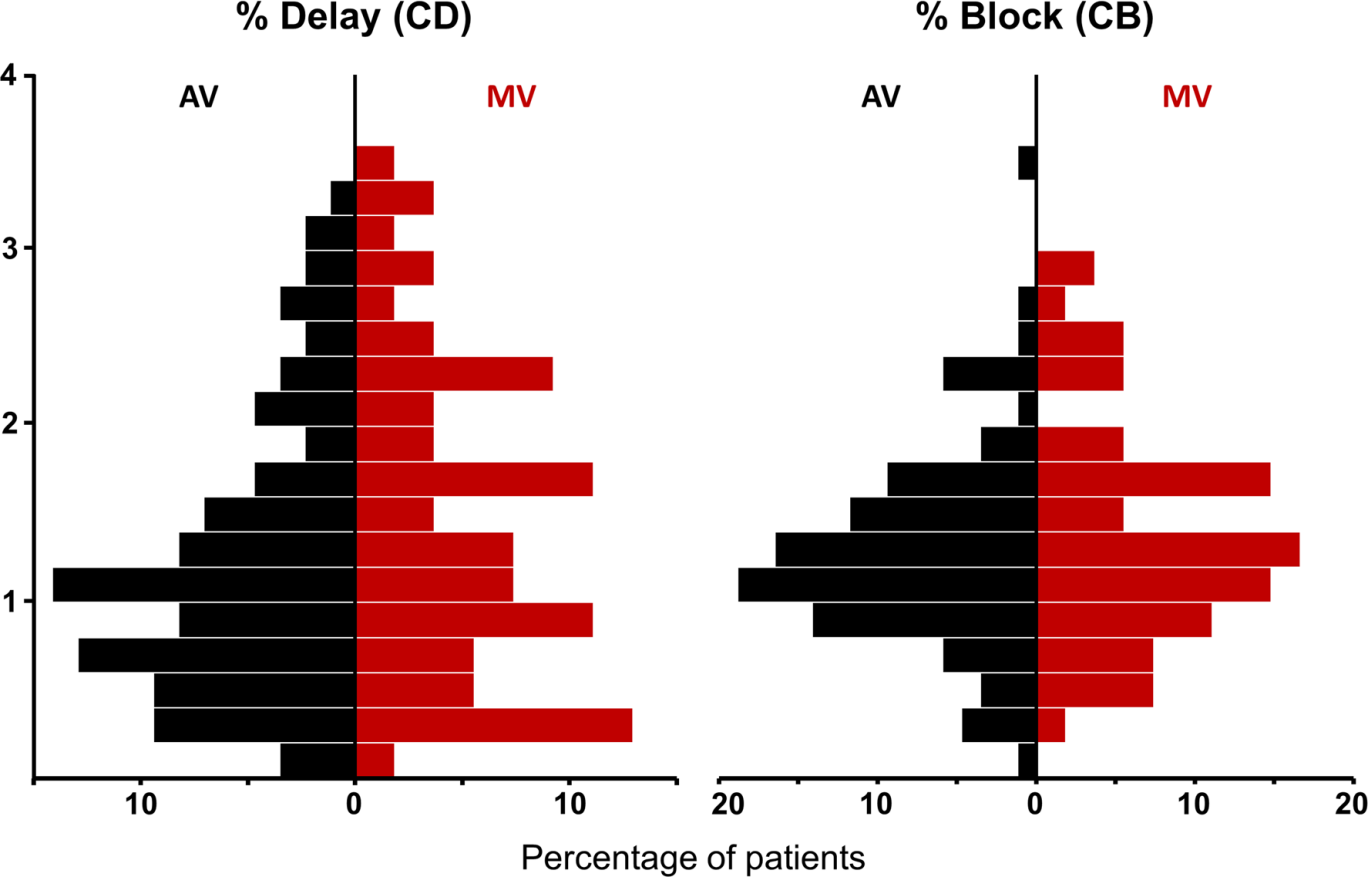
Overall conduction delay and block in valvular heart disease. Histograms of the amount of conduction delay and block (CD/CB) of the entire atrial surface in patients with aortic valve (AV) and mitral valve (MV) disease

A certain amount of both CD and CB was present in (nearly) all of the patients during sinus rhythm and the prevalence ranged respectively between 0.17–3.4% (median 1.26%) and 0–3.58% (median 1.15%) of the atrial surface. Figure 2 demonstrates the degree of CD and CB of the entire atria in patients with AV and MV disease. There was no difference in the atrial amount of CD/CB between patients with AV or MV disease. Both maximal peak gradient of patients with only aortic valve stenosis (*n* = 64, 78 ± 23 mmHg) and cycle length were not correlated to the total amount of CD and CB (*p* = 0.30 and *p* = 0.93).

### Atrial Distribution of Conduction Delay and Block

**Fig. 3:**
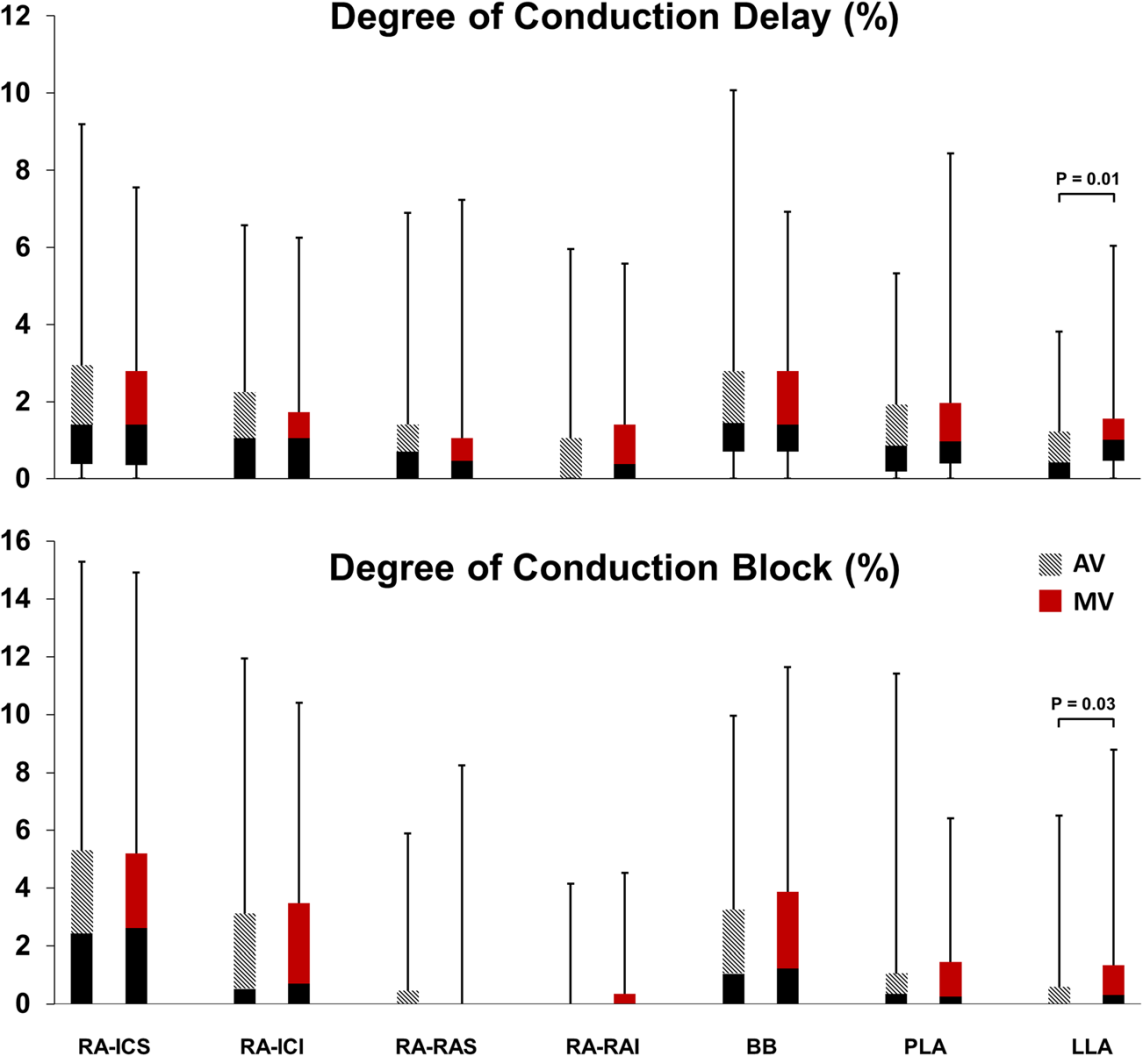
Conduction delay and block per atrial area. Boxplots of the average percentage of conduction delay and block (CD/CB) in each area in patients with AV and MV disease (error bars indicate minimum and maximum, box indicates Q1-median-Q3). The right atrial intercaval superior area (RA-ICS) has the highest amount of CB (*p* < 0.001, significant after Bonferroni correction). Patients with MV disease tend to have more CD/CB at the lateral left atrium (LLA). RA = right atrium; ICS/I = intercaval superior/ inferior; RAS/I = right appendage superior/inferior; BB = Bachmann’s bundle; PLA = posterior left atrium; LLA = lateral left atrium

Figure 3 demonstrates the average degree of CD and CB in each area for both groups. In Table 2, the maximal degrees of CD and CB for each area are shown. The largest difference in CD/CB between patients with AV and MV disease was seen at the lateral left atrial/LAA area. The maximal degree of CD at the lateral left atrium was higher in patients with MV disease and there was an overall trend towards more CD/CB at this site in the MV disease group. The average and maximal degree of CB was highest at the superior intercaval/terminal crest area of the right atrium (respectively 2.5% and 6.6%/cm^2^, all *p* ≤ 0.001). Long lines of CB are often seen towards the posterior area of the right atrium, also in two out of three of the youngest patients in our study group (21–24 years).

**Table 2:** Prevalence and maximal degree of CD and CB per area

		All			AV			MV			
		Prevalence (% patients)	Max degree (%/cm^2^)		Prevalence (% patients)	Max degree (%/cm^2^)		Prevalence (% patients)	Max degree (%/cm^2^)		*P* value^*^
RA-ICS	CD	78	4.1	(0–20.8)	79	4.2	(0–17.4)	77	4.0	(0–20.8)	0.391
	CB	73	6.6	(0–34.7)	74	6.3	(0–30.6)	72	6.9	(0–34.7)	0.635
RA-ICI	CD	70	2.8	(0–13.9)	72	2.8	(0–13.9)	66	2.9	(0–11.1)	0.578
	CB	55	2.1	(0–22.5)	53	2.1	(0–22.5)	59	2.8	(0–22.5)	0.696
RA-RAS	CD	63	1.4	(0–13.0)	65	1.4	(0–13.0)	59	1.4	(0–9.7)	0.489
	CB	32	0	(0–22.2)	38	0	(0–22.2)	24	0	(0–14.7)	0.138
RA-RAI	CD	53	1.4	(0–15.3)	47	0	(0–13.9)	62	1.5	(0–15.3)	0.118
	CB	20	0	(0–12.5)	15	0	(0–10.3)	26	0	(0–12.5)	0.086
BB	CD	84	4.2	(0–18.1)	82	4.2	(0–18.1)	87	4.2	(0–13.9)	0.847
	CB	70	3.5	(0–22.2)	71	2.8	(0–20.8)	67	4.2	(0–22.2)	0.630
PLA	CD	81	4.2	(0–18.8)	79	4.2	(0–18.8)	85	4.7	(0–18.1)	0.380
	CB	58	2.0	(0–23.6)	58	2.1	(0–23.6)	59	1.8	(0–19.6)	0.975
LLA	CD	75	2.8	(0–23.6)	69	2.8	(0–12.5)	85	4.2	(0–23.6)	0.001^†^
	CB	50	0	(0–20.8)	45	0	(0–20.8)	58	1.8	(0–17.4)	0.037

*P value of maximal degree of CB/CD between AV and MV patients. ^†^Significant after Bonferroni correction; *CD* conduction delay; *CB* conduction block; *RA* right atrium; *ICS/I* intercaval superior/ inferior; *RAS/I* right appendage superior/ inferior; *BB* Bachmann’s bundle; *PLA* posterior left atrium; *LLA* lateral left atrium

### Relation of Conduction Disorders with Pre- and Postoperative AF

Table 3 illustrates the relation and differences in clinical and electrophysiological parameters between patients with and without preoperative AF. AF patients were older, had more left atrial enlargement (> 45 mm), total CD/CB, and more CD/CB at Bachmann’s bundle and the lateral left atrium. The strongest parameter which correlated with preoperative AF was CD/CB at Bachmann’s bundle (r_rb_ = 0.40, *p* < 0.001) followed by age (r_rb_ = 0.31, *p* = 0.006).

**Table 3:** Correlation between a history of AF and clinical and electrophysiological characteristics

	No AF *N* = 101	AF *N* = 38	*P* value	Correlation coefficient
Age, yr [IQR]	68 [12]	73 [11]	0.006	0.31
Hypertension (%)	58	45	0.161	
Diabetes mellitus (%)	14	21	0.301	
LA > 45 mm (%)	22	47	0.003	0.25†
LV function (N-R-M %)	72 - 19 - 9	79 - 16 - 5	0.401	
CD/CB (average % [IQR])				
Total	2.3 [2.0]	2.7 [2.3]	0.044	0.22
RA-ICS	4.7 [6.6]	4.5 [5.0]	0.277	
RA-ICI	2.5 [5.1]	1.1 [5.7]	0.183	
RA-RAS	0.5 [1.8]	0.8 [1.8]	0.718	
RA-RAI	0.4 [1.4]	0.4 [1.3]	0.456	
BB	2.3 [4.4]	5.9 [6.4]	0.000*	0.40
PV	1.6 [2.3]	1.8 [3.2]	0.125	
LA	1.0 [1.9]	1.8 [2.5]	0.009	0.29

*Significant after Bonferroni correction; † Phi coefficient; *AF* atrial fibrillation; *IQR* interquartile range; *LA* left atrium; *LV* left ventricular; *N-R-M* normal-reduced-moderate; *CD* conduction delay; *CB* conduction block; *RA* right atrium; *ICS/I* intercaval superior/ inferior; *RAS/I* right appendage superior/ inferior; *BB* Bachmann’s bundle; *PLA* posterior left atrium; *LLA* lateral left atrium

Postoperative continuous rhythm registrations of 121(87%) patients were available and analyzed for occurrence of early postoperative AF. Postoperative AF occurred in 52%. Presence of total amount of atrial CD and CB in the upper quartile, preoperative AF, left atrial enlargement, left ventricular dysfunction, hypertension, and diabetes did not predict postoperative AF; only age was a limited risk factor for developing postoperative AF (OR 1.071, CI 1.023–1.122, *p* = 0.004). Neither univariate nor multivariate logistic regression including CD and CB in the upper quartile per area separately showed associations between electrophysiological parameters and postoperative AF.

## Discussion

High-resolution mapping of the atria in patients with valvular heart disease demonstrated a high and heterogeneous occurrence of disturbances in electrical conduction during sinus rhythm. The highest prevalence was seen at the high right atrium. The overall degree of CD and CB did not differ between patients with AV and MV disease. However, the lateral left atrium demonstrated a higher maximal degree of CD per 1 cm^2^ in patients with MV disease. Preoperative AF was most strongly correlated with conduction disorders at Bachmann’s bundle and age.

### Conduction Block at the High Right Atrium

Both groups demonstrated a high prevalence and degree of CB at the high right atrium which is not specific for patients with left-sided valvular heart disease and is also observed in patients with coronary artery disease [8]. The high right atrium is characterized by the sinoatrial node area and fractionated atrial electrograms are common at this site in patients with sinus node dysfunction [9]. However, likely CB seen at this site is not caused by sinus node disease with advancing age, but anatomically determined. Even young patients in this study had long lines of CB lateral to the site of first activation. Previous studies have demonstrated that the sinus node is surrounded by arteries and connective tissue protecting the node from external electrical influences and that action potentials of the sinus node are conducted to atrial myocardium via specific exit pathways located superiorly and inferiorly. Lateral conduction towards the atrial septum, however, is blocked by the insulating tissue [10, 11]. Furthermore, the neighboring terminal crest is known for anisotropic conduction properties and slow conduction parallel to the crest which may also contribute to the observed CB at the high right atrium [12].

### Conduction Disorders in AV Versus MV Disease

Both AV and MV disease have been associated with changes in the myocardial structure of the atria due to the altered hemodynamic effects [13, 14]. Structural remodeling can alter atrial electrophysiology and predispose to development of atrial tachyarrhythmias. The higher incidence of AF in MV disease suggests the presence of a higher degree of atrial remodeling in these patients. Roberts-Thomson et al. indeed demonstrated a larger amount of functional delay in conduction during pacing at the posterior left atrial wall in patients with MV disease compared to AV disease [15]. However, these differences were not present during sinus rhythm. We have investigated the entire atrial surface and identified the lateral left atrium as a location with increased conduction delay in patients with MV disease.

### Atrial Fibrillation and Conduction Disorders During Sinus Rhythm

In sinus rhythm, more CD was observed at the left posterior wall in MV patients with persistent AF than without AF [16]. In our study, patients with MV disease had more conduction disorders at the lateral left atrium and a higher prevalence of AF. However, preoperative AF was most strongly correlated with conduction disturbances at Bachmann’s bundle. Teuwen et al. has recently found that a high amount and long lines of CB at Bachmann’s bundle during sinus rhythm predisposes for early postoperative AF in patients with coronary artery disease [17]. The highly organized structure and anisotropic features could leave Bachmann’s bundle more vulnerable to structural remodeling and disturbances in conduction that can even be identified during sinus rhythm. The myocardial strands crossing the right and left atrial roof are not enclosed by fibrous tissue and may perhaps be easily disrupted by stretch due to overload of either atrium [18]. The histological study of Becker et al. demonstrated in fact that structural continuity of Bachmann’s bundle is often compromised, especially in patients with AF [19]. Conduction disorders at Bachmann’s bundle have been proposed to be a measure of more general atrial electrical pathology [20]. However, we found that conduction disturbances in other areas during sinus rhythm are not related to AF.

### Study Limitations

The routine preoperative echocardiograms did not allow for a retrospective sampling of exact dimensions. This parameter was therefore limited to the used cut-off dimension. The atrial septum cannot be reached by epicardial mapping and is therefore not included in this study. The atrial areas are recorded in a sequential manner due to technical restrictions which may cause some overlap between recordings.

### Clinical Relevance

This study is the first to describe conduction disorders in high-resolution of the entire atrial surface during sinus rhythm in patients with valvular heart disease. It demonstrated that left atrial overload in MV disease also translates to more conduction disorders at the lateral left atrium during sinus rhythm and that Bachmann’s bundle is most affected in MV and AV patients with AF. These findings help to further understand the structural damage caused by valvular heart disease that alters electrical conduction and most likely creates susceptibility for AF.

## Conclusions

Excitation of the atria during sinus rhythm is heterogeneously disrupted in patients with AV and MV disease; however, in MV disease, more conduction disorders are present at the lateral left atrium. The occurrence of AF in presence of valvular heart disease is associated with increased conduction disturbances in sinus rhythm at Bachmann’s bundle. Further research is necessary to determine the role of Bachmann’s bundle and lateral left atrium in the occurrence of AF in patients with valvular heart disease.

## Abbreviations

AF: Atrial fibrillation
AV: Aortic valve
CB: Conduction block
CD: Conduction delay
MV: Mitral valve

## References

[1] Darby AE, Dimarco JP (2012). Management of atrial fibrillation in patients with structural heart disease. Circulation.

[2] Verheule S, Wilson E, Everett T, Shanbhag S, Golden C, Olgin J (2003). Alterations in atrial electrophysiology and tissue structure in a canine model of chronic atrial dilatation due to mitral regurgitation. Circulation.

[3] Kumar S, Teh AW, Medi C, Kistler PM, Morton JB, Kalman JM (2012). Atrial remodeling in varying clinical substrates within beating human hearts: relevance to atrial fibrillation. Progress in Biophysics and Molecular Biology.

[4] Iung B, Baron G, Tornos P, Gohlke-Barwolf C, Butchart EG, Vahanian A (2007). Valvular heart disease in the community: a European experience. Current Problems in Cardiology.

[5] Sauter HJ, Dodge HT, Johnston RR, Graham TP (1964). The relationship of left atrial pressure and volume in patients with heart disease. American Heart Journal.

[6] van der Does LJ, Yaksh A, Kik C (2016). QUest for the Arrhythmogenic Substrate of Atrial fibRillation in Patients Undergoing Cardiac Surgery (QUASAR study): rationale and design. Journal of Cardiovascular Translational Research.

[7] Yaksh A, van der Does LJ, Kik C (2015). A novel intra-operative, high-resolution atrial mapping approach. Journal of Interventional Cardiac Electrophysiology.

[8] Lanters EA, Yaksh A, Teuwen CP (2017). Spatial distribution of conduction disorders during sinus rhythm. International Journal of Cardiology.

[9] Centurion OA, Fukatani M, Konoe A (1992). Different distribution of abnormal endocardial electrograms within the right atrium in patients with sick sinus syndrome. British Heart Journal.

[10] Fedorov VV, Schuessler RB, Hemphill M (2009). Structural and functional evidence for discrete exit pathways that connect the canine sinoatrial node and atria. Circulation Research.

[11] Fedorov VV, Glukhov AV, Chang R (2010). Optical mapping of the isolated coronary-perfused human sinus node. Journal of the American College of Cardiology.

[12] Becker R, Bauer A, Metz S (2001). Intercaval block in normal canine hearts: role of the terminal crest. Circulation.

[13] Anne W, Willems R, Roskams T (2005). Matrix metalloproteinases and atrial remodeling in patients with mitral valve disease and atrial fibrillation. Cardiovascular Research.

[14] Kim SJ, Choisy SC, Barman P (2011). Atrial remodeling and the substrate for atrial fibrillation in rat hearts with elevated afterload. Circulation. Arrhythmia and Electrophysiology.

[15] Roberts-Thomson KC, Stevenson IH, Kistler PM (2008). Anatomically determined functional conduction delay in the posterior left atrium relationship to structural heart disease. Journal of the American College of Cardiology.

[16] Roberts-Thomson KC, Stevenson I, Kistler PM (2009). The role of chronic atrial stretch and atrial fibrillation on posterior left atrial wall conduction. Heart Rhythm.

[17] Teuwen CP, Yaksh A, Lanters EA (2016). Relevance of conduction disorders in Bachmann’s bundle during sinus rhythm in humans. Circulation. Arrhythmia and Electrophysiology.

[18] Ho SY, Anderson RH, Sanchez-Quintana D (2002). Gross structure of the atriums: more than an anatomic curiosity?. Pacing and Clinical Electrophysiology.

[19] Becker AE (2004). How structurally normal are human atria in patients with atrial fibrillation?. Heart Rhythm.

[20] van Campenhout MJ, Yaksh A, Kik C (2013). Bachmann's bundle: a key player in the development of atrial fibrillation?. Circulation. Arrhythmia and Electrophysiology.

